# Preliminary Evidence for Neuronal Dysfunction Following Adverse Childhood Experiences: An Investigation of Salivary MicroRNA Within a High-Risk Youth Sample

**DOI:** 10.3390/genes15111433

**Published:** 2024-11-04

**Authors:** Adam T. Schmidt, Steven D. Hicks, Becca K. Bergquist, Kelsey A. Maloney, Victoria E. Dennis, Alexandra C. Bammel

**Affiliations:** 1 Department of Psychological Sciences, Texas Tech University, Lubbock, TX 79409, USA; 2 Center for Translational Neuroscience and Therapeutics, TTUHSC, Lubbock, TX 79409, USA; 3 Department of Pediatrics, Penn State College of Medicine, Hershey, PA 17033, USA; shicks1@pennstatehealth.psu.edu; 4 Department of Pediatrics, University of Tennessee Health Sciences Center, Memphis, TN 38163, USA

**Keywords:** adverse childhood experiences, microRNA, neuroinflammation, stress, gene expression, adolescents, mental health, minority youth

## Abstract

**Background/Objectives**: Adverse childhood experiences (ACEs) are potent drivers of psychopathology and neurological disorders, especially within minoritized populations. Nonetheless, we lack a coherent understanding of the neuronal mechanisms through which ACEs impact gene expression and, thereby, the development of psychopathology. **Methods**: This observational pilot study used a novel marker of neuronal functioning (brain-derived micro ribonucleic acids, or miRNAs) collected via saliva to explore the connection between ACEs and neuronal gene expression in 45 adolescents with a collectively high ACE exposure (26 males and 19 females of diverse races/ethnicities, with six cumulative ACEs on average). We aimed to determine the feasibility of using salivary microRNA for probing neuronal gene expression with the goal of identifying cellular processes and genetic pathways perturbed by childhood adversity. **Results**: A total of 274 miRNAs exhibited reliable salivary expression (raw counts > 10 in > 10% of samples). Fourteen (5.1%) were associated with cumulative ACE exposure (*p* < 0.05; *r*’s ≥ 0.31). ACE exposure correlated negatively with miR-92b-3p, 145a-5p, 31-5p, and 3065-5p, and positively with miR-15b-5p, 30b-5p, 30c-5p, 30e-3p, 199a-3p, 223-3p, 338-3p, 338-5p, 542-3p, and 582-5p. Most relations remained significant after controlling for multiple comparisons and potential retrospective bias in ACE reporting for miRNAs with particularly strong relations (*p* < 0.03). We examined KEGG pathways targeted by miRNAs associated with total ACE scores. Results indicated putative miRNA targets over-represented 47 KEGG pathways (adjusted *p* < 0.05) involved in neuronal signaling, brain development, and neuroinflammation. **Conclusions**: Although preliminary and with a small sample, the findings represent a novel contribution to the understanding of how childhood adversity impacts neuronal gene expression via miRNA signaling.

## 1. Introduction

Adverse childhood experiences (ACEs; e.g., child maltreatment; parental death or incarceration; and witnessing community or domestic violence) are known, potent risk factors for psychopathology in adolescents and adults and are exacerbating, if not driving, recent increases in psychiatric symptoms and emergency room visits for mental health disorders among adolescents throughout the world [1,2,3,4]. A substantial research base links adverse childhood experiences to multiple systemic and neurological diseases within adults, including cardiovascular disease, diabetes, multiple sclerosis, sleep disorders, and dementia including Alzheimer’s disease [5,6,7,8,9,10]. However, in addition to their well-known impacts on adult functioning, ACEs also result in increases in psychopathology among children and adolescents [11,12,13,14]. It remains unclear if ACEs alter cognitive functioning, emotion perception, susceptibility to other stressful life events, or some combination of these factors [11,15,16,17]. Moreover, adolescence marks a dynamic period of brain growth, including substantial increases in white matter development, cognitive changes, and hormonal maturation [18,19,20,21]. Other research suggests that disruptions to the normal processes of brain development occurring during adolescence can permanently alter the trajectory of cortical development, resulting in long-term behavioral outcomes [11,22,23,24,25,26,27]; however, the extent to which ACEs alter adolescent brain development is unclear [16,28].

Adverse childhood experiences are associated with changes in gene expression and function, including changes to genes associated with immune system activity, inflammation, and stress reactivity [16,17,29,30,31,32]. Despite the robust connection between ACEs and neurological and psychopathological conditions and between ACEs and altered gene expression, the brain mechanisms connecting ACEs to these outcomes remain unclear. Failure to understand this connection severely curtails the development of new pharmacological interventions and precision medicine approaches in psychiatry and neurology. Thus, there is a critical need to understand neuronal responses to ACE exposure.

Micro ribonucleic acids (miRNAs) are small, non-coding segments of RNA that regulate gene expression, including gene expression within the central nervous system (CNS) related to neuroinflammation, microglial activation, apoptosis, oxidative stress, and neuronal homeostasis and development [33,34,35,36]. A robust animal literature indicates brain-derived miRNA expression alters CNS gene expression following chronic early life stress [37,38], exposure to a stressful/traumatic stimulus [39], viral infection [36], and chronic stress [40,41,42]. Research with human participants suggests miRNAs can alter gene expression following chronic psychosocial stress [43]), relate to changes in genes involved with immune activation following trauma exposure, and prefigure genetic alterations in stress-related psychopathology and substance use disorders [44,45,46]. A recent study by Van der Auwera and colleagues [47] demonstrated that changes in miRNA expression relate to changes in genes involved in stress regulation in adults exposed to trauma in childhood. Despite these promising indications, no previous work has examined these issues within a vulnerable youth population, examined a wide range of miRNAs, or used a broad measure of ACEs that can capture a wide array of adversities.

Traditionally, the invasive procedures needed to collect miRNA samples (i.e., blood draws and lumbar punctures) made this research untenable with child populations. However, recent breakthroughs in non-invasive miRNA harvesting and analysis using salivary samples holds promise for addressing this critical knowledge gap. Neurons (including cranial nerves in the oropharynx) release miRNAs within exosomes as a mechanism for influencing gene expression and cellular processes and can be readily harvested via saliva collection [48,49,50,51]. The majority of non-coding RNAs (including microRNAs) in saliva are exosomal (rather than cellular [48]). Research indicates saliva contains a large amount of neuron- and glia-derived exosomes with miRNA cargo. These miRNAs (and other ncRNAs) are readily harvested by painless salivary swabs [51,52,53]. MiRNAs are extremely stable in saliva and can remain stored at room temperature for an extended period of time [52,53]. Other research indicates a substantial overlap between saliva and brain miRNA expression. Previous investigations in severe pediatric head injury demonstrated substantial consistency between salivary miRNA and miRNA obtained from cerebrospinal fluid [53]. These saliva miRNAs demonstrated enrichment for brain-related processes, and their concentrations were correlated with participant cognitive and behavioral symptoms [53]. 

To our knowledge, no studies have explored the connections between salivary miRNA concentrations and altered neuronal gene expression following ACE exposure within an adolescent sample. This gap represents a deficiency in our understanding of the neuronal responses to ACEs. Failure to clarify these cellular mechanisms retards progress toward identifying possible new therapeutic targets and decreases the ability to identify children at highest risk for negative outcomes. The current observational, cross-sectional study sought to identify candidate miRNAs that are associated with gene expression within the CNS and may be responsive to ACE exposure within a sample of underserved youth. Youth involved with the juvenile justice system represent an ideal population to investigate the role of child adversity in development because of their extremely high rate of ACE exposure (in excess of 90% as compared to 25–37% in the general population [54,55,56] and because of other contextual risk factors such as poverty, structural racism, and discrimination that may augment the impact of ACEs [11,57]. The working hypothesis was that miRNAs associated with inflammation broadly and neuroinflammation specifically would be related to overall ACE exposure within our sample of justice-involved youth (JIY).

## 2. Materials and Methods

### 2.1. Objectives

All procedures were approved by the Texas Tech institutional review board (Texas Tech University Human Research Protection Program, approval #IRB2019-485, approved 17 June 2019). The current study followed STROBE guidelines for cross-sectional observational research [58] applicable to pilot investigations. Written and verbal informed consent was obtained from caregivers at the time of recruitment and written and verbal informed assent was obtained from the adolescent participants at the time of testing. Caregivers and participants were told their participation was entirely voluntary and they could withdraw from the study at any time or choose to not answer any questions with which they felt uncomfortable. Both caregivers and adolescent participants provided their consent/assent for their deidentified data to be used for research purposes, including the publication of general findings based upon aggregate data. The data presented in the current study were obtained exclusively for the purposes of research and were not part of any clinical services (e.g., assessments, interventions, educational planning, etc.) provided to the participants, their families, or other professionals working with the youth.

### 2.2. Participants

The initial sample consisted of 48 total participants; missing data were handled using listwise deletion for a final sample of 45 participants for the current investigation. Thus, salivary miRNA samples were obtained from 45 JIY on probation (26 males, 19 females; *M*_age_ = 14.4; age range years: 12–17). Of the sample, 33.3% were Hispanic/Latino, 27.6% were Black, and 11.1% were White. Exclusionary criteria included physical disability prohibiting participation in study procedures, a history of intellectual disability diagnosis, and severe mental illness (i.e., psychotic and bipolar disorders). There were no additional exclusions based on other psychiatric diagnoses (e.g., autism, major depression, generalized anxiety disorder, conduct disorder, attention deficit hyperactivity disorder, etc.). Participants received community service hours toward their probation for their participation. However, participation was completely voluntary, and youth and their caregivers were free to withdraw from the study at any time without affecting their relationship with the juvenile justice facility. Based on previous research [52], this sample size was deemed adequate for a preliminary investigation.

### 2.3. Procedures

Participants were recruited from a juvenile justice facility in a medium-sized urban county in Northwest Texas between 1 September 2019 and 30 June 2022. In order to minimize the possibility of selection bias, all potentially eligible individuals were offered the opportunity to participate in the current investigation. Trained graduate researchers approached families during their hearings at the juvenile court. Researchers would not approach families displaying obvious signs of distress and would ask for permission to present information about the study prior to discussing study details. Researchers then explained the study to families (caregivers and the youth) who expressed interest in hearing about the research, and families were provided the opportunity to ask questions about the study. At the time of recruitment, caregivers provided written and verbal consent and contact information and completed a demographic questionnaire. Researchers contacted the caregivers who consented for their children to participate in the study to confirm eligibility and schedule time for the assessment and collection of saliva. Assessment took place in private rooms at the juvenile justice facility or within the laboratory of the principal investigator of the project (ATS). Researchers obtained written and verbal assent from the participating youth before proceeding with the assessment. Salivary samples were not collected, and no questionnaires were completed by the youth until and unless written and verbal assent were obtained. Participants did not consume food or beverages for at least 30 min prior to providing the salivary sample. Salivary samples were collected at the beginning of the session, followed by completion of the questionnaires. 

### 2.4. Measures

#### 2.4.1. Adverse Childhood Experiences

Adverse childhood experiences—which include direct and indirect trauma exposure as well as other childhood adversities—were assessed via the Adverse Childhood Experiences Questionnaire Teen Self-Report [59] ACE-Q Teen SR. The ACE-Q Teen SR is a 19-item self-report assessment of cumulative ACE exposure for youth aged 13 to 19, including abuse, neglect, and household dysfunction. These 19 items are separated into two sections. Section 1 assesses the 10 items included in the original ACE study [6], and Section 2 assesses an additional 9 items related to early life stressors (e.g., “You have been in foster care”, “You have had a serious medical procedure or life-threatening illness”). Instead of responding to each individual item, participants read each item and responded to each section with a total number of adversities experienced without identifying which items they were endorsing. This measure is preferred and is commonly used when working with vulnerable populations [60,61], as using a deidentified count of ACE exposure has also been shown to yield more valid responding and endorsement of specific ACE categories such as exposure to sexual abuse [59]. This may be because using a composite measure of ACEs may preclude the need to make reports to child and family protective services if a specific ACE (e.g., sexual or physical abuse) is reported. Child and Family Protective Services reports were made if a child endorsed abuse or maltreatment during other portions of the evaluation or if such a significant number of ACEs were endorsed as to leave no doubt that a reportable incident had occurred. Self-report measures of ACEs in adolescent samples, such as the ACE-Q-Teen SR, have high test–retest reliability [62,63]. No specific reliability or validity index information is currently available for the ACE-Q Teen SR [64], although the sum number of adversity experiences reduces the need for high internal consistency across items since it is not treated as a unidimensional scale. 

#### 2.4.2. Control Measures

To help control for retrospective reporting bias of ACEs, participants also completed the internalizing subscale from the Behavior Assessment System for Children, Third Edition (BASC-III;) to assess overall levels of internalizing symptoms such as anxiety, depression, and somatic concerns. The BASC-III is a broad-band measure of psychopathology and behavioral symptoms. For the current study, we used the Internalizing Problems scale as a control variable to account for the possibility that individuals who endorse a history of ACE exposure may be more likely to experience higher levels of internalizing symptoms [65]. For ages 12 to 14, the internal consistency coefficient of the BASC-III Internalizing composite scale is 0.96, and it is 0.97 for youth ages 15–18. Test–retest reliability of internalizing scale for adolescent report: r = 0.89; corrected r = 0.86. BASC-III scores are highly correlated with BASC-II scores and scores on other self-reports of psychiatric symptoms.

#### 2.4.3. miRNA Expression

Salivary samples for miRNA analysis were collected via expectoration in a non-fasting state, after an oral tap water rinse, using the CP-190 nucleic acid stabilization kit (DNA Genetic; Ottawa, ON, Canada). As previously described [53], this protocol increases the accuracy and reduces bias of the obtained miRNA samples [66]. Saliva samples were stored at room temperature in a secure laboratory facility of the PI prior to being shipped for analysis. Salivary RNA was extracted using the Oragene RNA purification protocol and the RNeasy mini column (Qiagen, Germantown, MD, USA). The yield and quality of the RNA samples were assessed using the Agilent Bioanalyzer. Following library construction, multiplexed samples were run on an Illumina HiSeq instrument at a targeted read depth of 10 million reads per sample (Illumina, San Diego, CA, USA). Reads were aligned to the human genome in Partek Flow using the Bowtie 2 algorithm. Mature miRNA levels within each sample were quantified using miRBase 22. The miRNAs with raw counts below 10 for greater than 10% of samples were excluded. A Deseq normalization technique with sum-scaling was applied. To evaluate the physiologic relevance of miRNAs that displayed relationships with ACE measures, target pathways were determined in DIANA miRPath v3 software [67] using the micro-T-cds algorithm, with a confidence threshold of 0.80 and an FDR-corrected *p*-value threshold of 0.05. The Kyoto Genes and Genomes (KEGG) pathways with mRNA representation exceeding that expected by chance alone were defined using a Fisher’s exact test (adjusted *p* < 0.05). Relationships between miRNAs of interest and their target KEGG pathways were visualized using a heatmap with hierarchical clustering.

### 2.5. Data Analysis Plan

All analyses were conducted with SPSS v. 28.0. Because this was a pilot investigation and we did not have any established correlations or other preexisting data with which to estimate a sample size calculation, all participants with complete data were included in the analyses. Our sample size of 45 is consistent with other pilot investigations of miRNA expression in adolescence [52]. Associations between miRNA and cumulative ACE exposure were assessed with Pearson correlation coefficients. Findings with *p*-values less than 0.05 were considered significant, and *p-*values between 0.05 and 0.1 are reported as trends. Comparisons between miRNA and high (≥4) versus low (<4) levels of ACE exposure were performed with between-subjects multivariate analysis of variance (ANOVA) tests. Additionally, comparisons were also conducted while controlling for internalizing symptoms of psychopathology using between-subjects multivariate analysis of covariance (ANCOVA). MicroRNAs were selected for comparisons if their Pearson correlation coefficient had a *p*-value less than or equal to 0.01 with the traditional ACE variable (i.e., 10 ACEs). False discovery rates (FDRs) were used to correct for multiple comparisons with a rate set at 0.10 [68]. The *F* values, degrees of freedom, and significance levels are reported for each miRNA with a significant or trending correlation. As noted above, missing data were handled using listwise deletion. 

## 3. Results

### 3.1. ACE Prevalence

Participants experienced 3.4 ACEs on average (*SD* = 2.5; range = 0–9), using the original 10 ACEs as a reference (e.g., physical abuse, sexual abuse, or physical or emotional neglect [6], with 88% of the sample experiencing at least one ACE. When examining additional life stressors, youth experienced 2.6 (*SD* = 1.2; range = 0–5) on average in addition to the 10 original ACEs (e.g., participation in the foster care system; experiencing a significant illness, injury, or medical procedure). Cumulatively, the sample had an average of 6 (*SD* = 3.2; range = 0–13) ACE exposures, with 97% of the sample experiencing at least one adverse experience, including maltreatment or life stressors. Additionally, 44% of the sample were exposed to a high level of ACEs (≥4; Kerker et al., 2015). In examining cumulative ACE exposure by participants’ sex, females, on average, experienced 6.7 ACEs (*SD* = 3.4, range = 0–12), and males experienced, on average, 5.4 ACEs (*SD* = 3.1, range = 0–13). 

### 3.2. miRNA Associations with Cumulative ACE Exposure

**Figure 1:**
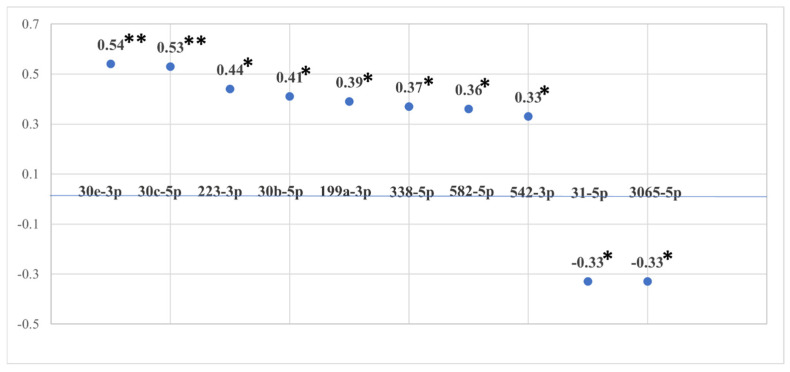
miRNAs with strongest correlations with total ACE scores. Note: the value attached to each data point represents the miRNA’s correlation with total ACE scores using r values. Two asterisks are used to denote correlations significant at the *p* < 0.01 level. One asterisk is used to denote correlations significant at the *p* < 0.05 level.

There were 274 miRNAs with reliable salivary expression (raw counts greater than 10 in greater than 10% of samples), and 14 (5.1%) demonstrated significant associations (*p* < 0.05; [R] ≥ 0.31) with cumulative ACE exposure. See Figure 1 for a depiction of the 10 strongest correlations between various miRNAs and the total ACE score (with stronger correlations being farther from the line, which represents a correlation value of 0). MicroRNAs 92b-3p, 145a-5p, 31-5p, and 3065-5p were negatively associated with cumulative ACE exposure, whereas 15b-5p, 30b-5p, 30c-5p, 30e-3p, 199a-3p, 223-3p, 338-3p, 338-5p, 542-3p, and 582-5p were positively associated. Further, results suggested correlations between many miRNAs and traditional ACEs related to child maltreatment (e.g., miR-15b-5p, 30b-5p, 30c-5p, 30e-3p, 186-5p, 199a-3p, 200a-5p, 223-3p, 338-5p, 338-3p, 365a-3p, 532-5p, 542-3p, 548-3p, 582-5p, and 3065-5p). ACEs related to other adverse experiences not captured by the traditional 10 ACEs included miR-30a-3p, 186-5p, 223-3p, 31-5p, 320a-3p, and 324-3p (The COVID-19 pandemic was added as an ACE variable to determine if there would be meaningful changes to overall ACE exposure and miRNA expression for participants tested during the pandemic (N = 18). Results indicated negligible differences that did not significantly change the interpretation of total ACE exposure with miRNA expression).

As JIY are prone to experience significantly more ACEs compared to the general adolescent population [11,57], a between-subjects multivariate analysis of variance was performed between miRNA and high versus low levels of ACE exposure (high = ≥4, low = <3; [13,69]). MicroRNAs were selected if they were correlated with the traditional ACE variable at the *p* ≤ 0.01 level (i.e., miR-30b-5p, 30c-5p, 30e-3p, 199a-3p, 223-3p, 338-5p, 365-3p, and 582-5p). Additionally, false discovery rates (FDRs) were used to correct for multiple comparisons, with a false discovery rate set at 0.10 [68]. The results of evaluation assumptions of normality, linearity, and multicollinearity were satisfactory. Tests of homogeneity of variance–covariance matrices were evaluated at an α of α = 0.001 due to unequal sample sizes across groups, which indicated that homogeneity of covariance matrices across groups is assumed (Box’s M = 80.54; *F*[36, 5029.2] = 1.75, *p* = 0.004; [70]). The multivariate result was significant for ACEs (Wilk’s Λ = 0.50, *F*[8, 34] = 4.18, *p* = 0.001), indicating a difference in the level of miRNAs across high versus low ACE exposure. The univariate *F* tests showed there was a significant difference between high versus low ACE exposure for miR-30b-5p (*F* = 8.55, *p* = 0.01), 30c-5p (*F* = 12.50, *p* = 0.002), 30e-3p (*F* = 17.53, *p* < 0.001), 199a-3p (*F* = 16.67, *p* = 0.02), 223-3p (*F* = 19.30, *p* < 0.001), 338-5p (*F* = 4.66, *p* = 0.04), and 365-3p (*F* = 4.94, *p* = 0.04) using FDR-adjusted *p*-values. See Table 1 for these *F* values and significance levels, which describe the differences in miRNA levels for youth with high ACE exposure compared to youth with low ACE exposure. Also, see Table 2 for descriptive statistics for these miRNAs.

Additionally, to try to control for retrospective bias in over-reporting ACEs, we conducted a between-subjects multivariate analysis of covariance on ACE categories and the selected miRNA after controlling for self-reported internalizing scores on the BASC-III. Results of evaluation assumptions of multivariate normality were assessed with Shapiro–Wilk tests for each miRNA at the high and low levels of ACE exposure. A conservative α level of 0.10 indicated this assumption was met for each miRNA. Assumptions of linearity between the dependent variables within each group of the independent variable were assessed with scatterplot matrices with loess lines. Visual examination of each matrix determined that this assumption was met. Tests of homogeneity of variance–covariance matrices were evaluated at an α of α = 0.001 due to unequal sample sizes across groups, which indicated that the homogeneity of covariance matrices across groups is assumed (Box’s M = 81.71; *F*[36, 4970.4] = 1.77, *p* = 0.003; [70]. The multivariate result was significant for ACEs after controlling for internalizing scores (Wilk’s Λ = 0.50, *F*[8, 32] = 4.32, *p* = 0.001), indicating a difference in the level of miRNAs across high versus low ACE exposure irrespective of internalizing scores. See Table 3 for between-subjects test statistics, which describe the differences in miRNA levels for youth with high ACE exposure compared to youth with low ACE exposure while controlling for internalizing scores (with the bottom half of the table comprised of between-subjects test statistics describing the difference in miRNA levels for youth with high internalizing scores compared to youth with low internalizing scores).

**Figure 2:**
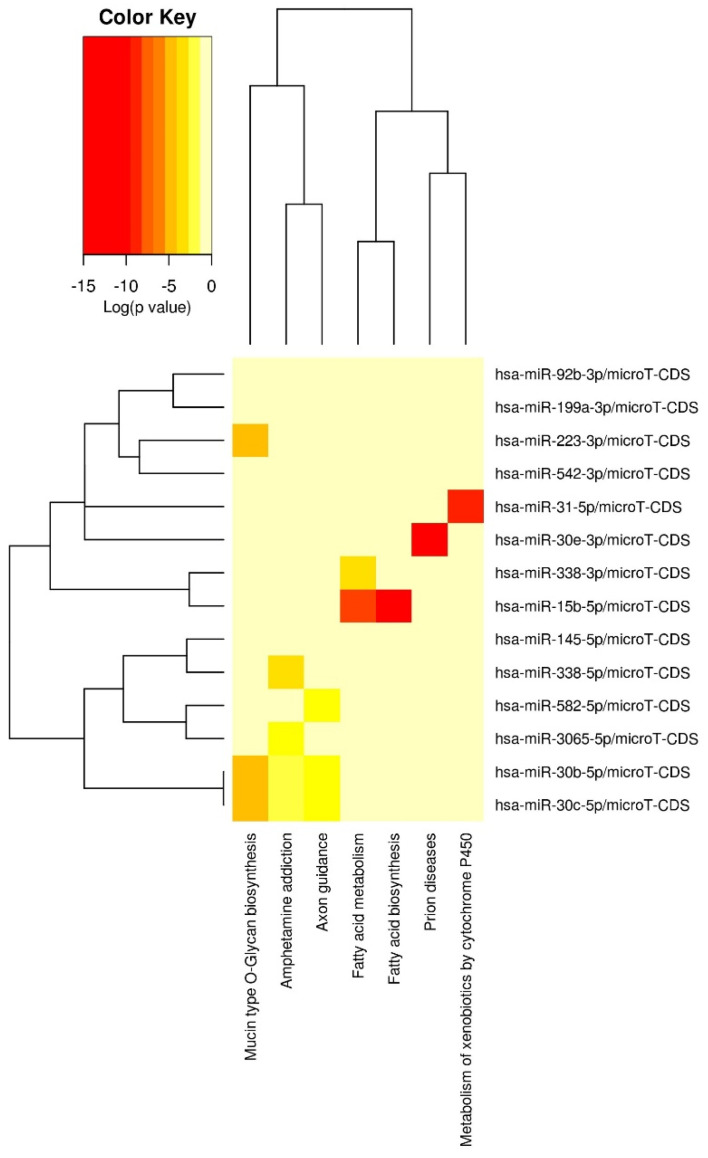
Heatmap of overlapping KEGG pathway targets for select microRNAs. Note: the heatmap displays all 14 candidate microRNAs, but only 7 pathways are pictured as these are the pathways with the absolute highest overlap; however, not all of these pathways are uniquely related to brain function, so they are not included in the broader discussion of the findings.

Finally, to determine the relevance of our findings to neuronal gene expression, we examined the KEGG pathways targeted by the miRNAs associated with the total ACE score. Results indicated putative miRNA targets over-represented 48 KEGG pathways (adjusted *p* < 0.05). Figure 2 depicts a heatmap of overlapping KEGG pathway targets between all 14 miRNAs and the seven pathways with the greatest overlap (also see Appendix A Table A1, which depicts KEGG pathways targeted by miRNAs associated with total ACE score). Among these physiologic pathways were several involving brain-related signaling (e.g., AMPK [*p* = 0.002, 59 genes, 13 miRNAs] and regulation of glutamatergic synapses [*p* = 0.002, 50 genes, 14 miRNAs]); brain development (e.g., WNT signaling [*p* = 0.002, 63 genes, 14 miRNAs] and axon guidance pathways [*p* = 0.002, 60 genes, 14 miRNAs]); and inflammation/neuroinflammation (e.g., TGF-β [*p* < 0.001, 38 genes, 14 miRNAs], mTOR [*p* < 0.001, 35 genes, 12 miRNAs], PI3K-Akt [*p* = 0.002, 138 genes, 14 miRNAs], and MAPK [*p* = 0.047, 98 genes, 14 miRNAs]). Notably, similar results were obtained when examining the significant miRNAs related to traditional ACEs as well as to ACEs related to household dysfunction.

## 4. Discussion

The goal of the current investigation was to determine if miRNAs regulating neuronal gene expression were related to exposure to child adversity with the long-term goal of identifying neuronal mechanisms linking ACEs to psychopathology, cognitive dysfunction, and neurologic disorders. We found compelling preliminary evidence that neuronally derived salivary miRNAs were related to both the 10 traditional ACEs as well as a broader list of 9 additional adverse experiences associated with household dysfunction, interpersonal violence, and discrimination. Our findings remained significant after correcting for multiple comparisons and controlling for self-reported internalizing symptoms. Notably, many of the significant miRNAs related to ACE exposure are linked to inflammatory/neuroinflammatory genetic pathways implicated in a variety of neurological and neurodevelopmental disorders (e.g., TGF-β, PI3K-Akt, and mTOR; [71]). Consistent with the investigation of Van der Auwera and colleagues [47], we showed that miR-30b and 30e were disrupted by ACE exposure. Our findings are also largely consistent with previous work showing that neuroinflammatory miRNAs are related to altered gene expression in various neurological disorders. For example, increases in miR-223-3p have been observed in animal models of traumatic brain injury [72,73] and in hippocampal tissue from human patients with a history of epilepsy secondary to tuberous sclerosis [74]. Other research associates upregulated miR-30b and 30e and downregulated miR-338-5p with neuropathology within Alzheimer’s disease [75,76,77] and miR-223-3p with peripheral nerve injury [78]. Of more relevance to the current study, elevated levels of miR-30e-3p, along with a proinflammatory phenotype, were observed in the ventral hippocampus of passive coping rats following a social defeat paradigm [79]. Other studies link miR-15b, 31-5p, and 199a upregulation and miR-200a downregulation more directly to neuroinflammation [80,81,82,83,84,85]. Research links ACEs to a proinflammatory phenotype [16,31]. Research associates some of the miRNAs altered in the current study with inflammation-related changes in gene expression. These include miR-223-3p in autism [86] and multiple sclerosis [37]; miR-30b-5p, 30c-5p, 186-5p, and 532-5p in Amyotrophic Lateral Sclerosis [87]; and miR-582-5p and miR-223-3p and inflammation related to environmental toxicity [88].

We found partial support for the findings of Van der Auwera and colleagues [47] who observed changes in miRNAs in adult samples following a history of self-reported child adversity. These authors used blood-derived miRNA collection, and most, but not all, of the 15 miRNAs they observed were available to us in the current study of saliva. Consistent with the findings of Van der Auwera and colleagues [47], we observed changes in miR-30b and 30e. However, none of the other miRNAs they investigated were significantly related to ACE exposure. MiR-30b and 30e are implicated in various neurological disorders, including traumatic brain injuries, Amyotrophic Lateral Sclerosis, Parkinson’s disease and Alzheimer’s disease [53,77,87,89]. The inconsistencies between the current study and the Van der Auwera study may be related to differences in participant age, measures of ACE exposure, biofluid of miRNA measurement, or method of RNA quantification.

The current investigation has several important limitations that may potentially limit generalizability and that should be addressed. First, the literature on miRNA expression in human conditions is clearly in its nascency and is plagued by small sample sizes and a lack of longitudinal investigations. The field is in need of findings such as those in the current study to be replicated across time and populations. Therefore, the current findings are best viewed as exploratory and require confirmation with larger, more diverse samples; longitudinal follow-ups; and controls for additional confounds. Second, retrospective self-reports of ACEs have been critiqued for potentially measuring current psychological distress as opposed to being an unbiased report of actual adversities experienced [13]. However, we controlled for current internalizing symptoms in our analyses to partly address this shortcoming of retrospective reporting, and our findings remained unchanged. Nevertheless, future studies would benefit from the addition of prospective reports of ACEs and/or official reports of child maltreatment to determine if our pattern of results can be replicated. Third, the present findings are limited by a lack of specificity on which individual ACEs were endorsed by participants. Although evidence suggests that this deidentified approach yields more valid reporting among an adolescent sample [59], future research would benefit from the use of measures where specific ACEs are endorsed and information on the frequency or severity of these experiences is also captured. This type of more detailed data would allow for a more granular understanding of the impacts of specific ACEs on the developing brain. Fourth, the current findings should be specifically replicated with other samples using QRT-PCR to analyze and confirm miRNA expression patterns in a new sample. Finally, although previous research indicates that brain-derived miRNAs are available via exosomes found in saliva [51,53]), the current findings are limited by a lack of brain-specific metrics (e.g., measures of cognition and/or neuroimaging). Nonetheless, the genes regulated by the altered miRNAs are involved with brain-related functions, and an abundance of human and animal studies link many of the altered miRNAs more directly to neuronal gene expression. Future studies need to examine how miRNA concentrations may relate to actual metrics of brain structure (e.g., gray and white matter volumes, specific white matter tracts, cortical surface area and thickness, etc.) and function as well as to performance on cognitive/neuropsychological tests (e.g., tests of executive function, attention, memory, etc.) to further validate the current findings. Likewise, exploring the role of individual miRNAs in psychiatric symptoms was beyond the scope of this initial investigation. As such, future studies should examine the connections between ACE exposure, miRNA concentrations, and psychiatric disorders to determine the practical relevance of the current findings for understanding the course and development of psychopathology and neurologic disease.

These limitations notwithstanding, this investigation is the first to examine the relations between salivary miRNA concentrations and changes in neuronal gene expression following ACE exposure, and to our knowledge, is the first to examine changes in neuronal gene expression using miRNA expression within a human adolescent sample. Although preliminary in nature, if validated with larger samples and longitudinal studies, the current findings may have implications for research and clinical practice. With regard to research implications, miRNAs each have multiple gene targets, and a better understanding of how ACEs impact miRNA expression may identify novel pathways and gene ensembles involved in an individual’s response to child adversity. More broadly, this study illustrates the potential role of microRNAs in regulating gene expression within the CNS and contributes to our rudimentary understanding of how microRNAs may impact brain development and, potentially, how these epigenetic markers may regulate gene expression in response to environmental inputs. Although the current results do not have immediate implications for clinical practice, they suggest the impacts of child adversity may already be present and exerting an effect at a cellular/neuronal level during adolescence. Thus, they underscore the need for additional screening and emphasize the opportunities and importance of early interventions shown to minimize the impact of ACEs, such as mindfulness and stress reduction, sleep hygiene training, nutritional and exercise interventions [90], and, for individuals already experiencing psychological symptoms, empirically supported psychological therapies such as cognitive behavioral therapy and trauma-informed interventions [91,92]. In the future, these findings may open up new pharmacological avenues for treatment following chronic ACE exposure and/or acute traumatic events. For example, understanding the cellular processes involved in the neuronal responses to ACEs could open up new treatment targets, such as disrupting neuroinflammatory pathways. Moreover, although extremely early in development, researchers have been exploring the therapeutic value of miRNA mimics and inhibitors for treating brain injuries, degenerative brain diseases, and psychiatric disorders [93,94]. Medications could potentially be used preventatively to target specific families of miRNAs demonstrated to exacerbate neuroinflammation or other stress pathways in individuals presenting with high levels of ACE exposure (e.g., children placed in foster care, individuals repeatedly exposed to community violence). This strategy of prevention would mark a substantial departure from conventional symptom management approaches and may hold promise for reducing the considerable economic, social, and health burdens of ACEs.

In conclusion, results suggest a role for miRNAs in regulating gene expression within the CNS following ACE exposure and provide evidence for miRNA involvement in coordinating the neuronal responses to ACEs. Although beyond the scope of the current investigation to determine, it is interesting to speculate that neuroinflammation may be occurring at low, chronic levels, eventually leading to psychiatric disorders over time or with repeated ACE exposures. Our findings are bolstered by their theoretical and empirical consistency with prior human and animal research and by the overlap in inflammatory and neuroinflammatory genetic pathways targeted by the identified miRNAs. They provide unique insights into the neuronal mechanisms perturbed by ACE exposure within human participants. Further, if replicated, they lay the initial foundation for novel therapeutic approaches to mitigate the long-term impacts and health consequences of child maltreatment and other forms of child adversity.

## Figures and Tables

**Table 1:** Tests of between-subjects effects across ACE categories representing most significant microRNA correlations.

Source	Dependent Variable	Mean Square	F	Sig. ^a^	Partial Eta Squared
ACEs	miR-223-3p	1.10	19.32	<0.001	0.32
	miR-30e-3p	0.45	17.53	<0.001	0.30
	miR-30b-5p	0.32	8.55	0.01	0.17
	miR-30c-5p	0.31	12.50	0.002	0.23
	miR-365a-3p	0.14	4.94	0.04	0.10
	miR-582-5p	0.14	2.58	0.11	0.05
	miR-199a-3p	0.28	6.67	0.02	0.14
	miR-338-5p	0.17	4.66	0.04	0.10

Note: ^a^ = FDR-adjusted *p*-values.

**Table 2:** Means and standard deviations of most significant microRNAs.

MicroRNA	Mean	Standard Deviation
miR-223-3p	0.24	0.29
miR-30e-3p	0.12	0.19
miR-30b-5p	0.02	0.24
miR-30c-5p	0.074	0.21
miR-365a-3p	−0.09	0.21
miR-582-5p	0.10	0.23
miR-199a-3p	0.18	0.22
miR-338-5p	0.12	0.20

**Table 3:** Tests of between-subjects effects across ACE categories, controlling for internalizing scores and representing most significant microRNA correlations.

Source	Dependent Variable	Mean Square	F	Sig. ^a^	Partial Eta Squared
ACEs	miR-223-3p	1.27	23.17	<0.001	0.05
	miR-30e-3p	0.42	16.39	<0.001	0.01
	miR-30b-5p	0.21	5.74	0.03	0.04
	miR-30c-5p	0.30	11.61	0.005	0.00
	miR-365a-3p	0.08	2.90	0.10	0.07
	miR-582-5p	0.06	1.20	0.27	0.05
	miR-199a-3p	0.30	7.47	0.01	0.00
	miR-338-5p	0.14	3.83	0.07	0.00
Internalizing Scores	miR-223-3p	0.12	2.19	0.37	0.37
	miR-30e-3p	0.01	0.59	0.70	0.29
	miR-30b-5p	0.06	1.73	0.38	0.12
	miR-30c-5p	0.01	0.33	0.74	0.22
	miR-365a-3p	0.09	3.28	0.56	0.06
	miR-582-5p	0.12	2.38	0.52	0.03
	miR-199a-3p	0.00	0.07	0.90	0.16
	miR-338-5p	0.00	0.03	0.85	0.09

Note: ^a^ = FDR-adjusted *p*-values. Note: The top half of the table examines between-subjects test statistics, which describe the differences in miRNA levels for youth with high vs. low ACE exposure while controlling for internalizing scores. The bottom half of the table examines between-subjects test statistics for the differences in miRNA levels for youth with high vs. low internalizing scores (with no control variable).

## Data Availability

The data presented in this study are available on request from the corresponding author as the data also form part of an ongoing study.

## Appendix A

**Table A1:** KEGG pathways targeted by miRNAs associated with total ACE score.

KEGG Pathway	Sig. ^a^	Number of Genes	Number of miRNAs
Fatty acid biosynthesis	<0.001	6	7
Long-term depression	<0.001	34	12
Hippo signaling pathway	<0.001	68	14
Signaling pathways regulating pluripotency of stem cells	<0.001	71	14
Prion diseases	<0.001	8	8
TGF-β signaling pathway	<0.001	38	14
Proteoglycans in cancer	<0.001	88	14
Renal cell carcinoma	<0.001	36	11
Ubiquitin-mediated proteolysis	<0.001	64	14
mTOR signaling pathway	<0.001	35	12
Prostate cancer	0.001	45	12
Regulation of actin cytoskeleton	0.001	93	14
AMPK signaling pathway	0.002	59	13
Axon guidance	0.002	60	14
PI3K-Akt signaling pathway	0.002	138	14
Adherens junction	0.002	40	13
N-Glycan biosynthesis	0.002	21	14
WNT signaling pathway	0.002	63	14
Focal adhesion	0.002	91	14
Gap junction	0.002	38	14
Glutamatergic synapse	0.002	50	14
Pathways in cancer	0.002	160	14
Transcriptional misregulation in cancer	0.003	69	14
Adrenergic signaling in cardiomyocytes	0.003	63	14
Lysine degradation	0.003	23	13
FoxO signaling pathway	0.003	60	13
Glioma	0.003	30	14
2-Oxocarboxylic acid metabolism	0.005	9	9
cGMP-PKG signaling pathway	0.011	70	14
Melanoma	0.013	36	12
Fatty acid metabolism	0.015	18	12
ErbB signaling pathway	0.015	41	14
Rap1 signaling pathway	0.015	87	14
Protein processing in endoplasmic reticulum	0.017	67	13
Arrhythmogenic right ventricular cardiomyopathy (ARVC)	0.020	35	14
Bacterial invasion of epithelial cells	0.020	38	14
Choline metabolism in cancer	0.023	47	13
Thyroid hormone signaling pathway	0.026	51	14
Ras signaling pathway	0.028	89	14
Cell cycle	0.033	52	13
Oxytocin signaling pathway	0.039	65	14
Morphine addiction	0.039	37	14
MAPK signaling pathway	0.047	98	14
Cholinergic synapse	0.047	49	14
Amphetamine addiction	0.048	27	13
Endocytosis	0.049	80	14
Viral carcinogenesis	0.049	66	14
Endocrine and other factor-regulated calcium reabsorption	<0.050	17	12

Note: ^a^ = FDR-adjusted *p*-values.
